# Aerated Cement Slurry and Controlling Fungal Growth of Low-Cost Biomass-Based Insulation Materials

**DOI:** 10.1038/s41598-019-55626-5

**Published:** 2019-12-17

**Authors:** Areej T. Almalkawi, Parviz Soroushian

**Affiliations:** 1 Dept. of Civil and Environmental Engineering, Michigan State University, Michigan, USA; 2 Metna Corporation, 1926 Turner Street, Lansing, Michigan USA

**Keywords:** Environmental sciences, Environmental impact, Sustainability

## Abstract

Wood chips or particles as well as shredded straw offer desired thermal insulation qualities. When exposed to a humid environment, however, fungal growth on wood and straw is an important consideration. An experimental investigation was conducted in order to evaluate the effectiveness of a simple treatment in mitigating fungal growth on wood- and straw-based insulation. This treatment involved blending of wood chips or particles, or shredded straw with an aerated slurry which offers the potential to mitigate fungal growth on biomass by a combination of physical and chemical effects without imposing a weight penalty. Experimental results verified the effectiveness of this treatment in controlling fungal growth on wood and straw subjected to different moisture conditions.

## Introduction

Biomass ash-based insulation in the form of wood chips or ground wood offers desired thermal insulation qualities complemented with low cost, ready availability, and favorable ecological features. There are, however, concerns with the resistance to fire, moisture and fungus growth of such insulation. The work reported herein focuses on improvement of the resistance to fungus growth of low-cost biomass-based insulation comprising largely of wood chips or ground wood.

Fungal proliferation inside buildings can have adverse effects on the health of building residents^1–3^; it can also cause discoloration and degradation of construction^4–6^. Mold growth on conventional insulation materials is habitually related to moisture occurred from flooding, water condensation or plumping leaks^7^. Many research investigations have addressed the impact of dampness level on the tendency of construction materials to mold growth^8^. At relatively low levels of humidity, fungi do not tend to inoculate on the surfaces of building materials^9^. Relative humidity levels of 70% to 90% are sufficient to trigger fungal augmentation on construction materials^10,11^. Besides the relative humidity of the environment, the water holding capacity (WHC) and/or the equilibrium moisture content (EMC) of a particular building material are also important indicators of the potential for fungal growth on a material exposed to humid conditions^12,13^.

Building materials that are broadly categorized as “green” are becoming increasingly popular for indoor (and other) applications. Green indoor products are often promoted for low-emission, low-toxicity and recyclability attributes^14–16^. These qualities contrast those of the highly popular reconstituted wood products (e.g., oriented strandboard – OSB) used in interior building applications that are fabricated with such adhesives as urea formaldehyde that release harmful emissions and are prone to fungal growth.

Reconstituted wood products have realized their current prominence because they can make value-added use of wood strands/fibers/particles that are abundantly available at low cost. The work reported herein concerns the use of low-cost and abundantly available wood flakes or ground wood as insulation materials in building construction. These low-cost and abundant products would be viable as insulation materials as far as they complement viable insulation qualities with desired resistance to fungal growth as well as moisture and fire. The work reported herein concerns the insulation attributes and the resistance to fungal growth on wood flakes and ground wood. Low-cost treatment methods are also developed for achieving improved resistance to fungal growth.

Bio-based materials have been used in construction for centuries^17^. They can exhibit desired durability and may deteriorate under the action of microorganisms such as fungi and bacteria. The activity of such microorganisms depends mainly on environment factors (relative humidity and temperature), and also on the building material (substrate) characteristics such as hygroscopicity^18,19^. Bio-based materials may hold a potential source of nutrients for fungal and bacterial activity. Under similar environmental conditions, different bio-based materials may be affected differently by mold growth^20,21^. Their resistance against mold growth decides their suitability for construction application. This property is usually assessed in the critical moisture content that is amenable to fungal growth^22,23^.

Resistance of materials to mold growth can be enhanced by the addition of biocides. Boric acid and derived salts (borates) are widely used as biocide along with commercial insulation materials such as blowing cellulose^20,24^. Such substances have the dual-action as a biocide and as a fire retardant; they are viewed as a greener alternative to metal-based fungicides^25^, commonly considered in wood preservation; their toxicity is, however, scrutinized nowadays^26–28^. Another approach is the use nonbiocidal techniques for protection against fungi^20,29,30^. Bio-based insulation materials can be impregnated with water repellents, such as waxes^31^, or pretreated to reduce their hygroscopicity^32,33^. This work evaluated the effectiveness of impregnating bio-based thermal insulation materials with aerated cement slurry in order to improve their resistance to fungal growth in different humidity conditions.

## Materials

### Wood particles

**Figure 1:**
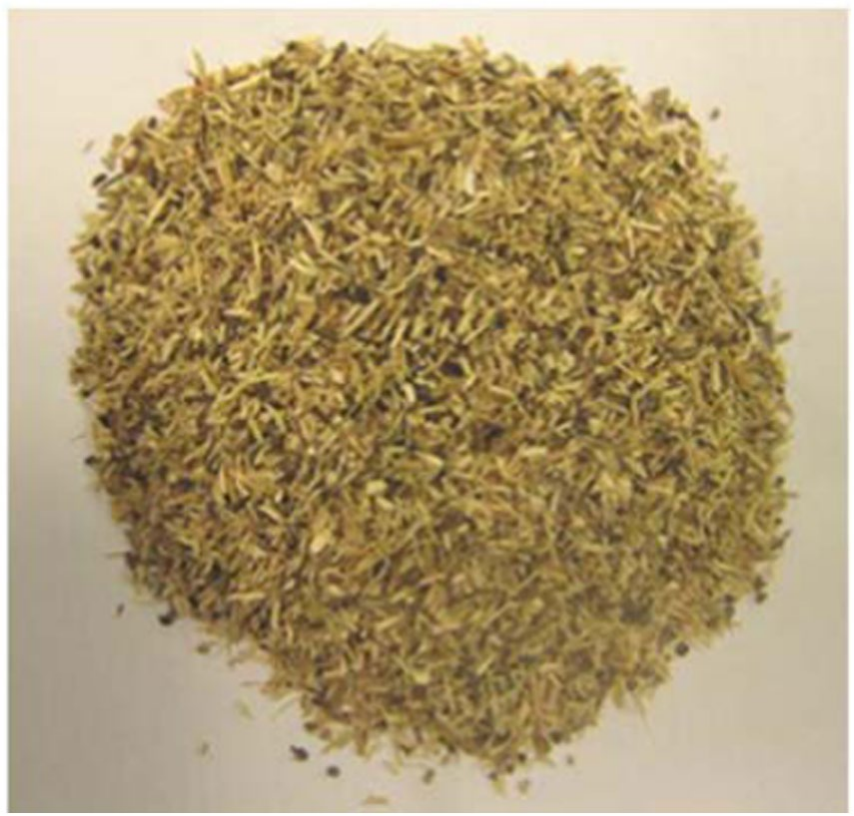
Fine wood particles.

**Figure 2:**
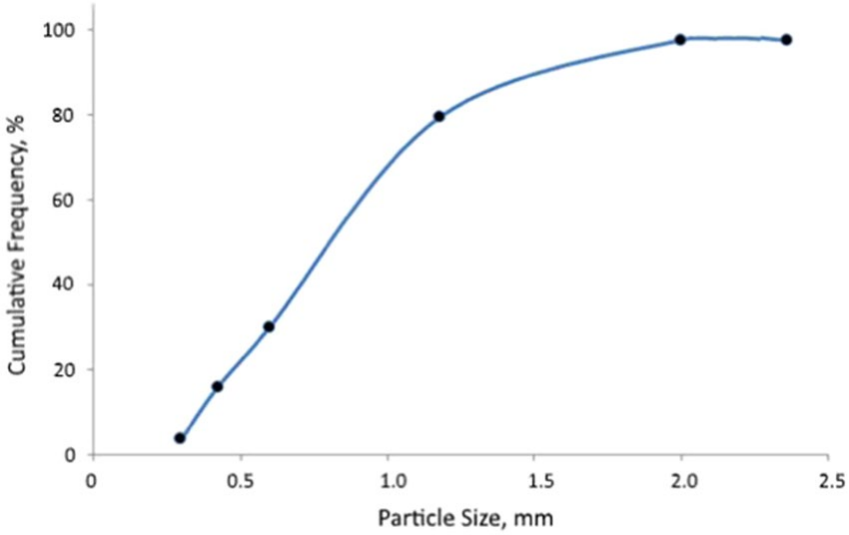
Particle size distribution of the ground wood insulation.

Wood branches collected locally (in air-dried condition) were ground using a hammer mill. The resulting wood particles were passed through a sieve with 2.36 mm opening. Figure 1 shows the appearance of the fine wood particles which were evaluated as insulation material. While these fine wood particles could be used for thermal insulation without any treatment, mitigation of microbial degradation would be a concern. Figure 2 shows the particle size distribution of the fine wood particles using sieve analysis method. The median particle size was about 0.8 mm, with particles ranging in size from 0.3 to 2.36 mm. The loosely packed fine wood particles had a bulk density of 0.291 g/cm^3^.

### Wood chips

**Figure 3:**
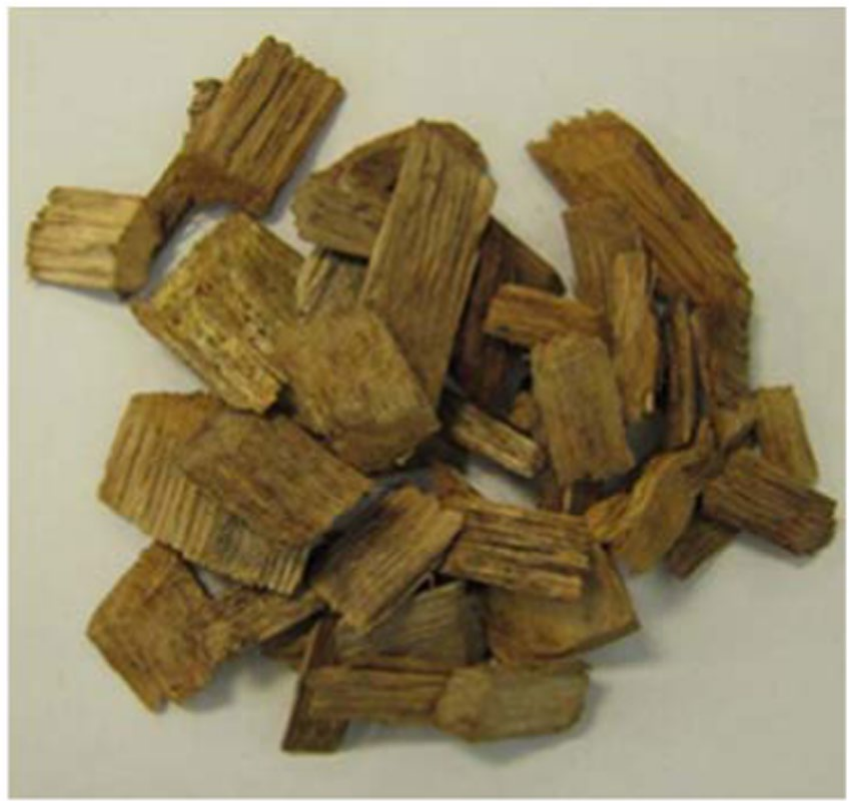
Wood chips.

**Figure 4:**
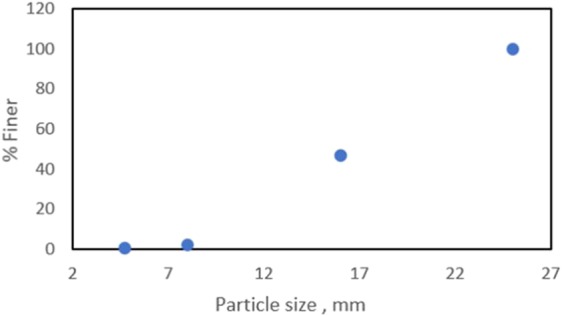
Particle size distribution of the wood chips insulation.

Wood chips with less than 25 mm particle size (Fig. 3) **Error! Reference source not found**. were also considered as sustainable insulation materials. The particle size distribution of these wood chips is presented in Fig. 4. The bulk density of these wood chips was 0.205 g/cm^3^.

### Shredded straw

**Figure 5:**
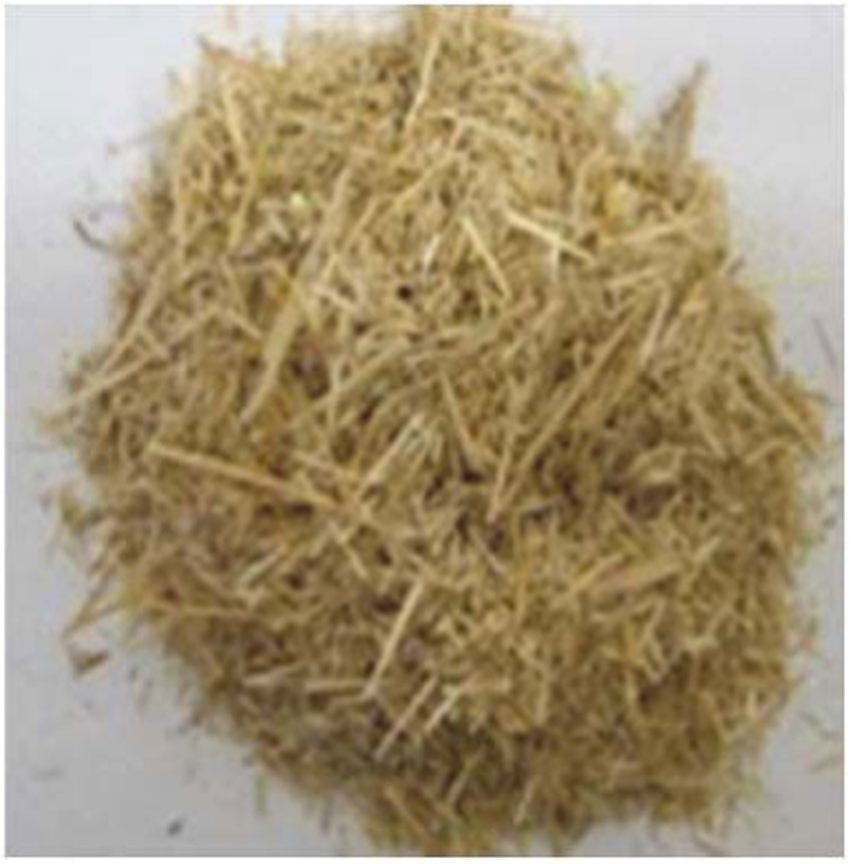
Shredded straw.

**Figure 6:**
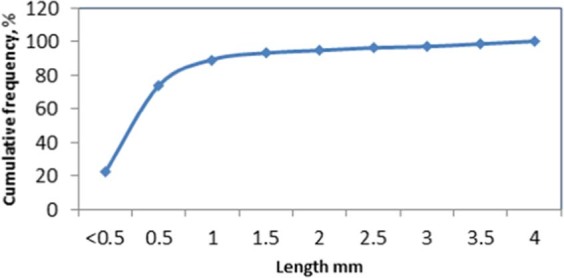
Length distribution of the shredded straw.

Shredded wheat straw (Fig. 5) with the length distribution shown in Fig. 6 was also used in this investigation. The bulk density of shredded straw was 0.610 g/cm^3^.

### Treatment of biomass with aerated slurry

**Figure 7:**
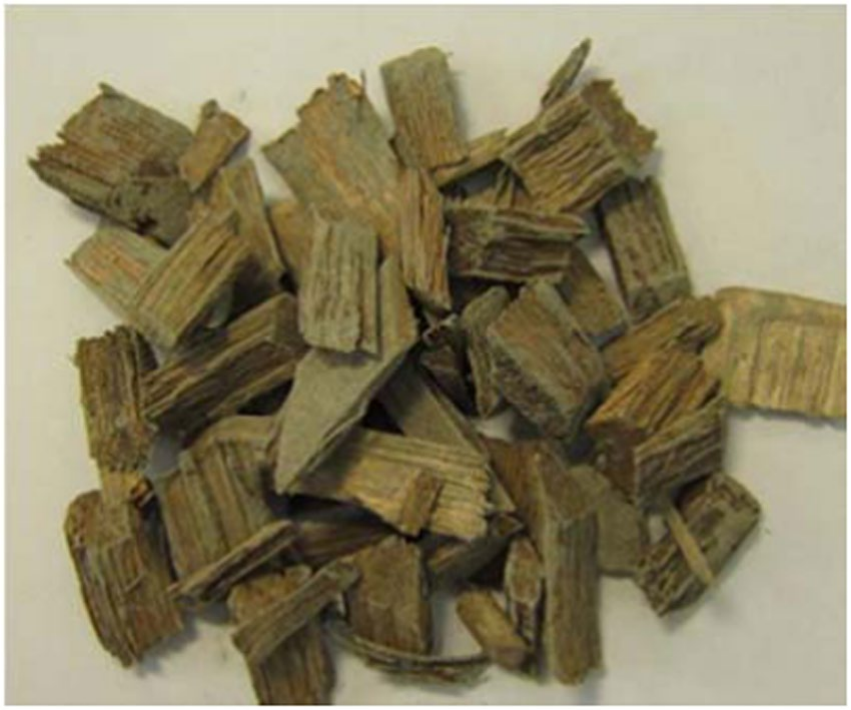
Wood chips treated with aerated cement slurry.

An aerated cement slurry was used for pretreatment of fine wood particles, wood chips and shredded straw in order to improve the resistance of biomass to fungal growth without imposing major weight penalties. The aerated slurry was prepared at a water/cement ratio of 0.6, with saponin used as the foaming agent. Saponin was first added to the mixing water and stirred at a rotational speed of 1,200 rpm to produce a stable foam. The foamed water was then mixed with cement in a planetary mixer over 12 minutes to produce a homogenous aerated slurry; the dosage of the foaming agent (saponin) was adjusted to produce (after curing and oven-drying of slurry) a bulk density of 0.75 g/cm^3^. Physical and mechanical properties of aerated slurry cement were studied in our previous works^34–38^. The aerated slurry was added to fine wood particles, wood chips or shredded straw at an aerated slurry-to-biomass weight ratio of 0.2 in a drum mixer. Figure 7 shows wood chips after treatment with aerated slurry. This treatment raised the bulk density of wood chips, finer wood particles and shredded straw from 0.291 to 0.295, from 0.205 to 0.214, and from 0.610 to 0.627, respectively.

The thermal conductivities of find wood particles and wood chips measured, prior to an after treatment with aerated cement slurry, are summarized in Table 1. When compared with the thermal conductivities of commercially available thermal insulation materials such as expanded polystyrene (Styrofoam), that is 0.033 W/m.K, fine wood particles and wood chips are observed to provide reasonable low thermal conductivities for use as thermal insulation in building construction. The thermal conductivity of shredded straw was more than three times those of fine wood particles and wood chips, which could be due to convective heat transfer of loose straw. Treatment of wood straw with aerated slurry slightly reduced its thermal conductivity, which could be due to a slight reduction in the convective heat transfer. This trend was opposite of that observed with fine wood particles and wood chips, which experienced a slight rise in thermal conductivity after treatment with aerated slurry.

**Table 1:** Thermal conductivities of fine wood particles and wood chips.

Specimen	Thermal conductivity, W/m.K
Fine wood particles	0.0516
Fine wood particles (treated)	0.0535
Wood chips	0.0638
Wood chips (treated)	0.0587
Shredded straw	0.196
Shredded straw (treated)	0.189

## Methods

### Water-holding capacity

The water-holding capacity of the insulation materials was measured by keeping triplicate samples (container sized 4 cm cube) in water as submerged condition till the saturation for flooding simulation purposes. The maximum water-holding capacity (WHC, %) of the samples was expressed as the mass ratio of water to dry materials in Equation.

$$WHC=({M}_{final}-{M}_{initial})/{M}_{initial}\times 100$$

where, M_initial_ is the initial weight of air-dried sample (g), and M_final_ is the weight of fully saturated sample (g). The mean and standard deviation values were computed for all triplicated samples.

### Equilibrium moisture content

The equilibrium moisture content of the insulation samples was measured according per ASTM C1498-04a (2004) and ASTM D2216-05 (2005). Samples of insulation materials (5 cm cube) were dried initially at 105 °C to reach a constant mass. Triplicated insulation materials kept in sealed condition in 48 L stainless-steel chambers with relative humidity (70%) and temperature range of (22–25 °C). A constant air exchange rate of 1 h^−1^ was maintained and passed through the chamber at relative humidity of 70%. The weight of each specimen was monitored over regular intervals for constant mass observation, the equilibrium moisture content was determined per the equation:

$$EMC=({M}_{final}-{M}_{initial})/{M}_{initial}\times 100$$

where, EMC is the equilibrium moisture content (%); where, M_initial_ is the initial weight of air-dried sample (g) and M_final_ is the weight of sample at equilibrium condition with the water vapor in air inside the chamber (g). the values averaged for all triplicated measurements.

### Artificial inoculation

**Figure 8:**
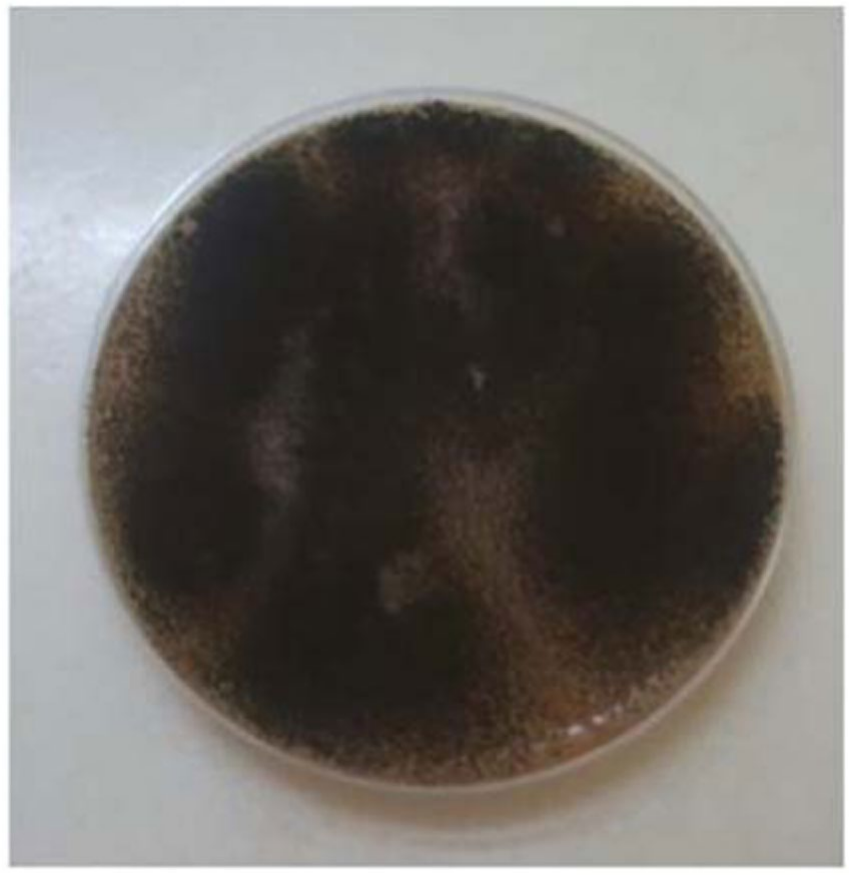
Fungi (Aspergillus) inoculated in a nutrient medium.

The aim of this investigation was to testify the vulnerability of insulation materials to augemntation by a model fungal species subjected to different nutrient sources at favorable temperature and humidity ranges. Aspergillus niger was chosen as the ideal fungus type for this indoor environment condition^2,39,40^. The A. niger strain was obtained from the American Type Culture Collection (Rockville, MD, USA) with designed number (ATCC 9642). Development of spore (conidia) inocula was conformed per the ASTM G21-96. A spore suspension was stimulated by augmenting the fungi for two weeks on V8 agar culture. 5 ml of a based wetting-agent solution (0.2% NaNO_3_, 0.05% KCl, 0.03% K_2_HPO_4_, 0.05% MgSO_4_, 0.02% KH_2_PO_4_, 0.01% Tween 80, 0.001% FeSO_4_) was poured in Petri dish, and the culture were kindly moved to a sterile bottle. The insulation materials were artificially inoculated by spraying 0.4 ml of suspension solution onto the specimens. The confined Petri dishes (Fig. 8) holding the wetted and inoculated materials were kept in a sterile bench at incubation conditions 25 °C for 28 days.

To evaluate the potential of fungal growth, the insulation materials were moisturized to three levels: ambient level, simulated rain, and the water holding capacity (WHC) level. The ambient moisture condition reached by subjecting the materials to 22 °C and 34–40% relative humidity for 24 hours. Simulated rain was produced by showering the specimens for 5 minutes with approximately 530 ml water followed by drying for 24 hours at ambient condition of 20 °C and 35–40% relative humidity. This process produced a moisture condition that in-between ambient and WHC and simulated at the same time an accidental wetting throughout construction building. The WHC condition was achieved by storing the samples in water for two days.

**Figure 9:**
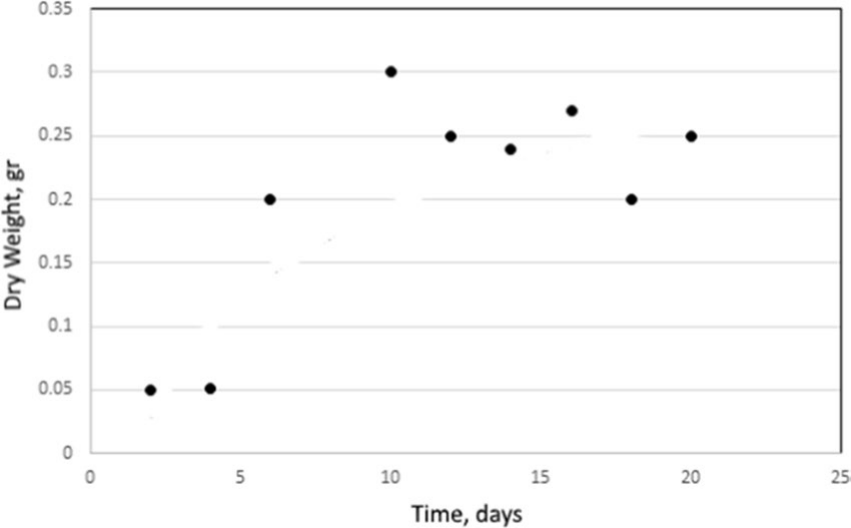
The growth curve of Aspergillus niger cultivated as batch cultures in nutrient medium (each data paint is the mean value of two measurements).

The weight gain of Fungi A. niger was monitored over the inoculation period. They exhibited rapid weight from 4 to 8 days in nutrient medium, after which they reached the stationary phase of incubation (Fig. 9).

Fungal growth on different insulation materials was visually detected on a weekly basis following the ASTM C1336 (96) procedures. Microscopic inspections were made when fungus growth was noted; the amount of growth was classified in categories using the following scale:

**Table:** 

No growth noticed microscopically	0
Microscopical cultivation existence	1
Microscopical cultivation coating all surface	2
Macroscopical (visible to naked eye) cultivation existence	3
Macroscopical cultivation covering 75% of the surface	4

An image processing tool (image J software) developed by the U.S. National Institutes of Health (NIH) was used to determine the surface area of the entire sample (A_s_) and that of the area contaminated with fungal growth (A_c_)^41–43^. The percentage of the surface covered by fungal growth (P_c_) was calculated as (Ac/As ×100) and was plotted versus the incubation time for each insulation material. The fungal growth rating was confirmed by microscopic observation (20X) and with the aid of a digital image (taken by a 16 Mgp Samsung camera).

### Compliance with ethical standards

This chapter does not contain any studies with human participants performed by any of the authors.

## Results and Discussion

### Water uptake

**Figure 10:**
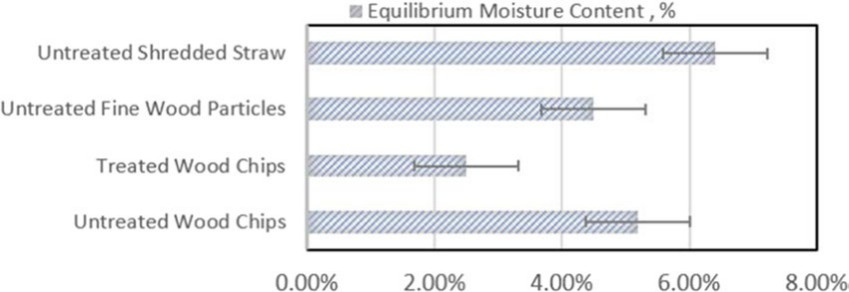
Equilibrium moisture contents of selected insulation materials.

The equilibrium moisture content (EMC) test results at 85–92% relative humidity and 20 °C temperature were presented in Fig. 10. EMC reflects a dynamic equilibrium, and changes with the relative humidity and temperature by the surrounding air. The EMC of the wood- and straw-based insulation materials ranged from 5 to 6%. Treatment of wood chips with aerated slurry reduced their equilibrium moisture content by about 50%.

Moisture contents exceeding 18% would encourage growth of fungi which were present in wood and straw as spores and may degenerate cellulose to create what is commonly referred to as “dry rot”. At moisture contents below 18%, fungi become dormant. If the moisture content exceeded a certain level, fungi may grow exponentially^44,45^.

**Figure 11:**
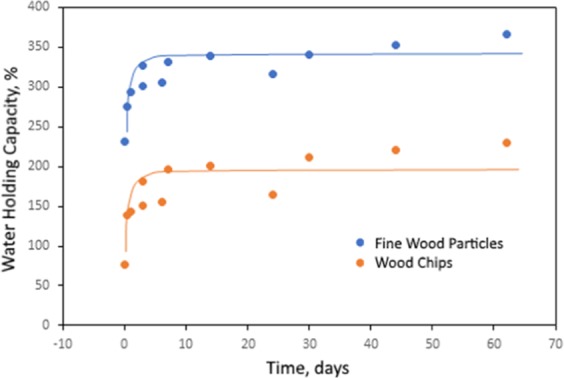
Water holding capacity test results for untreated wood ships and fine wood particles over a submersion period of 65 days.

The water holding capacity test results for untreated wood chips and untreated fine wood particles, measured over 65 days of submersion, were presented in Fig. 11. Fine wood particles had a water holding capacity of 366%, which was higher than that for wood chips (230%). Both fine wood particles and wood chips exhibited a tendency to immediately absorb some moisture.

### Visual and microscopic observations

**Figure 12:**
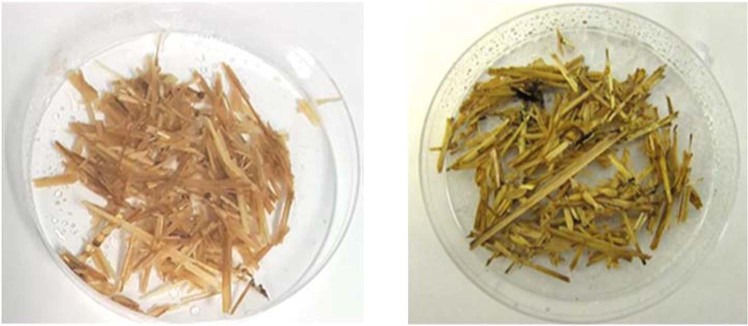
An example of visually notable fungus growth on untreated shredded straw that was submerged in water for 48 hours and then exposed to 21 days of fungal. (**a**) Untreated wood straw prior to (left), and 21 days after introduction of fungi (right).

Visual inspections were made to evaluate the susceptibility of bio-based insulation materials to fungal growth. An example of visually notable fungal growth on the biomass insulation materials was presented in Fig. 12. The test data summarized in Table 2 indicate that the trends in fungal growth on untreated wood chips and untreated shredded straw are similar. Treatment of wood chips with aerated slurry was highly effective in mitigating fungal growth for all the moisture conditions considered here. After four weeks, treated wood chips in ambient and simulated rain conditions did not exhibit any indications of fungal growth. It was only after four weeks that minor fungal growth could be detected on treated wood chips in the ‘water holding capacity’ moisture condition. In the case of treated shredded straw, fungal growth was not observed after 3 weeks of exposure in ambient and simulated rain conditions and was minor in the ‘water holding capacity’ moisture condition. In ambient condition fungal growth was not observed on treated shredded straw after 4 weeks; fungal growth was present on treated shredded straw after 4 weeks in simulated rain and ‘water holding capacity’ moisture conditions.

**Table 2:** Results of the visual examination of fungal growth on untreated and treated biomass insulation materials over time in different moisture conditions (ASTM C1338): ambient moisture (Amb), simulated rain (Rain), and water wetted to water holding capacity (WHC).

Average value of growth visible on surface of insulation materials												
	1 Week			2 Week			3 Week			4 Week		
	Amb	Rain	WHC	Amb	Rain	WHC	Amb	Rain	WHC	Amb	Rain	WHC
Untreated Wood Chips	0	0	0	0.5	1	1	0	1.5	2	2	3	2
Untreated Shredded Straw	0	0	0	0.5	1	1	0	1.5	2	2	3	2
Treated Wood Chips	0	0	0	0	0	0	0	0	0	0	0	0.5
Treated Shredded Straw	0	0	0	0	0	0	0	0.5	0.5	0	1	1

**Figure 13:**
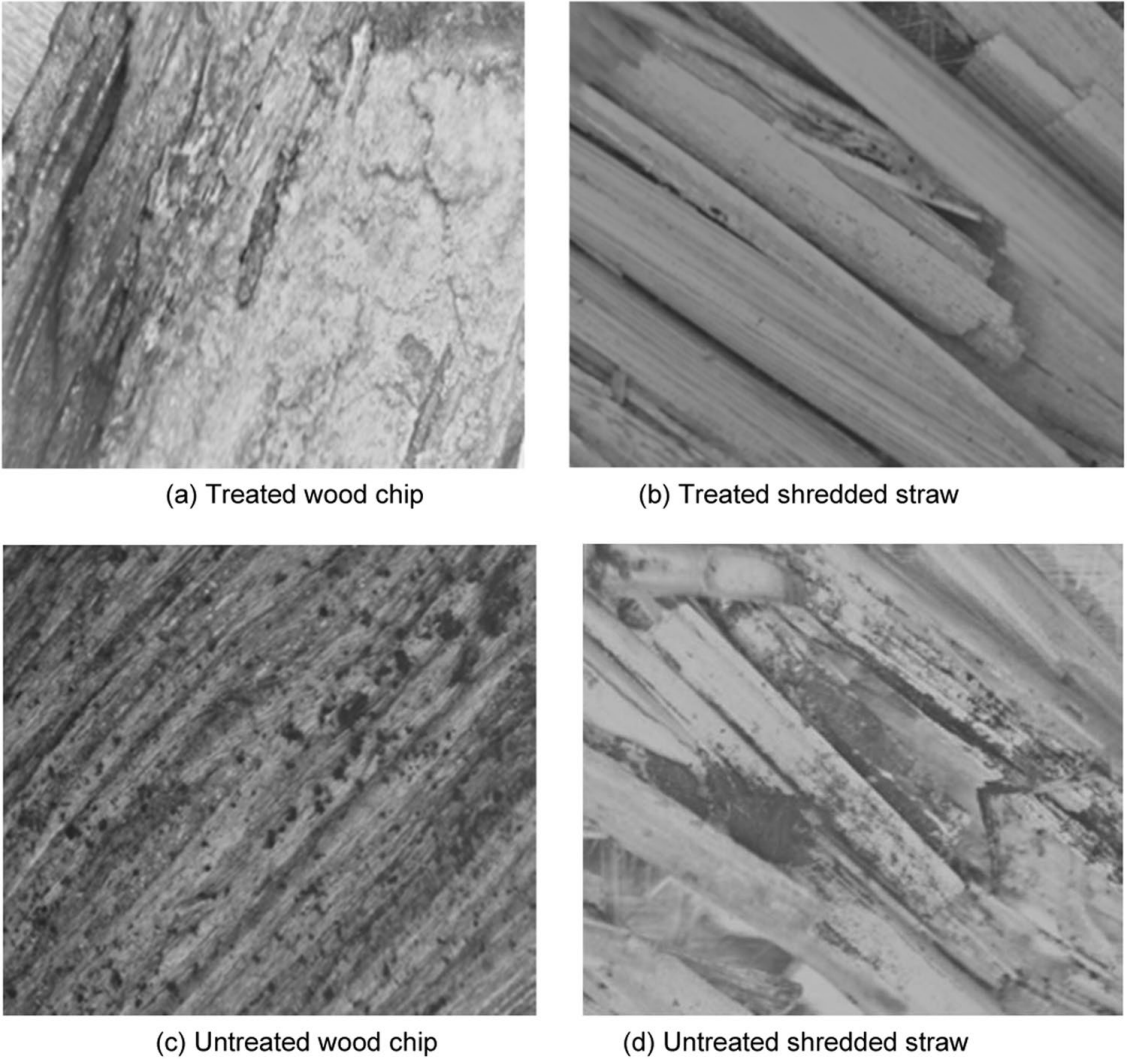
Optical microscope images of treated and untreated wood chips and shredded straw after 4 weeks of exposure to fungi in ambient moisture.

Optical microscope examination of treated wood chips (Fig. 13a) and treated shredded straw (Fig. 13b) after 4 weeks of exposure in ambient condition confirmed that treatment with aerated slurry was effective in mitigating fungal growth on these biomass insulation materials. For untreated wood chips (Fig. 13c) and untreated shredded straw (Fig. 13d), on the other hand, fungal colonization on surfaces was obvious under similar exposure conditions. Similar trends have been reported for cement-bonded wood particleboards made with eucalypt and ryber wood particles^46^.

### Natural inoculation

**Figure 14:**
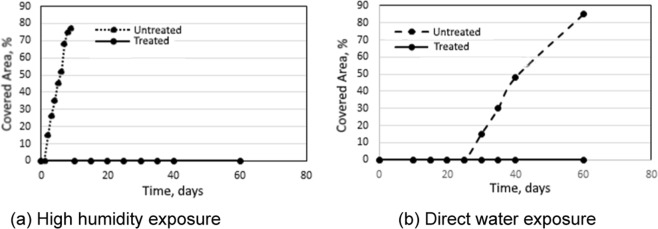
Fungal coverage of untreated and treated wood chips subjected to high humidity or direct water exposure prior to incubation.

Figure 14 showed the percent of surface area covered by fungal growth versus incubation time following natural inoculation with A. niger fungi. When untreated wood chips were subjected to high humidity prior to incubation, fungal growth was observed within the first week of incubation. When exposed directly to water, fungal growth on untreated wood chips occurred after a lag period of 25 days. This lag period can be explained by: (i) the need for moisture content to drop below a threshold level required to support fungal growth; and (ii) washing away of some naturally inoculated fungi during the submersion period. Fungal growth did not occur on treated wood chips irrespective of the pre-exposure to high humidity or directly to water.

The effectiveness of aerated slurry treatment as an effective means of mitigating fungal growth on the biomass ash-based insulation could be attributed to: (i) coating of biomass and filling of its pores with aerated slurry, which produces a physical barrier against access of fungi to biomass; (ii) the relatively high pH of aerated cement slurry (pH = 11); and (iii) the amenability of the partially cured aerated slurry to further curing upon exposure to moist conditions.

## Conclusions

Wood chips and particles as well as shredded straw are low-cost and abundantly available materials with desired thermal insulation qualities. Fungal growth on wood and straw in humid environments, however, is a concern. An experimental investigation was conducted in order to assess the merits of a simple and low-cost method of treating wood chips/particles and straw for control of fungal growth without while retaining their desired thermal insulation quality without imposing a weight penalty. The following conclusions were derived based on the test data generated in this investigation.

1. Treatment of wood chips/particles and shredded straw with aerated slurry effectively mitigates fungal growth in diverse moisture and fungal growth conditions.
2. Treatment of wood and straw with aerated slurry does not produce any notable changes in the thermal insulation and bulk density due to the relatively low bulk density of aerated slurry, and the use of a relatively low dosage of aerated slurry for treatment of wood or straw.
3. The effectiveness of aerated slurry against fungal growth on wood and straw can be attributed to: (i) its ability to effectively fill the surface pores and core the surfaces of biomass, producing a physical barrier against fungal growth; (ii) the relatively high pH of aerated cement slurry; and (iii) the amenability of the partially cured slurry on wood and straw surfaces to benefit from exposure to humid environments via further curing.
